# Cost utility and budget impact analysis of dexamethasone compared with bortezomib and lenalidomide for the treatment of second line multiple myeloma from a South African public health perspective

**DOI:** 10.1186/s12962-022-00399-4

**Published:** 2022-12-12

**Authors:** Lineo Marie Matsela, Susan Cleary, Thomas Wilkinson

**Affiliations:** 1 Health Economics Division, School of Public Health and Family Medicine, Faculty of Health Sciences, University of Cape Town, Cape Town, South Africa; 2 World Bank Group, Washington, DC USA

**Keywords:** Lenalidomide, Bortezomib, Relapsed/refractory multiple myeloma, Cost effectiveness, Second-line treatment

## Abstract

**Background:**

Multiple myeloma is an incurable haematological malignancy that is associated with a high probability of relapse. The survival of relapsed patients has been greatly improved by the development of novel drugs such as lenalidomide and bortezomib. We assessed the cost-effectiveness of these drugs as second-line treatment for relapsed/refractory multiple myeloma (RRMM) patients in the South African public health care system.

**Methods:**

We modelled 3 treatment strategies for second-line RRMM treatment: dexamethasone (standard of care), bortezomib (BORT) and lenalidomide plus dexamethasone (LEN/DEX) from the South African public health perspective. For each strategy we modelled a hypothetical cohort of relapsed/refractory multiple myeloma patients using a three-state Markov model over a 15-year time horizon. Efficacy and utilization data were obtained from the MM009/010 and APEX trials and external studies. Price and cost data were from local sources and presented in 2021 South African Rands. Outcomes were reported in quality adjusted life years (QALYs). Incremental cost effectiveness ratios (ICERs) were calculated for BORT and LEN/DEX and compared to a local cost-effectiveness threshold of R38 500 per DALY averted using the assumption that 1 DALY averted is equal to 1 QALY gained. A budget impact analysis was conducted to evaluate the financial impact of the introduction of BORT and LEN/DEX, respectively. Deterministic sensitivity analysis was undertaken to account for parameter uncertainties.

**Results:**

The modelled total costs of DEX, BORT and LEN/DEX were estimated to be R8 312, R234 996 and R1 135 323, respectively. DEX treatment provided 1.14 QALYs while BORT and LEN/DEX treatments provided 1.49 and 2.22 QALYs, respectively. The ICER of BORT versus DEX was R654 649 and that of LEN/DEX versus BORT was R1 225 542. Both BORT and LEN/DEX treatments were not cost-effective relative to a cost-effectiveness threshold of R38 500 per DALY averted. Both BORT and LEN/DEX significantly increase the 1 year budget-cost of RRMM treatment.

**Conclusion:**

Both BORT and LEN/DEX treatments are unlikely to be cost-effective strategies for second-line treatment of RRMM in South Africa. The results indicate that the drug prices of lenalidomide and bortezomib are key drivers of value for money. Price reductions could potentially make BORT more cost-effective.

## Background

Multiple myeloma is the second most common haematological malignancy in Sub-Saharan Africa [1]. In South Africa, it accounts for less than 1% of all cancers. It mainly affects older people above the age of 65 years, which is concerning given future population ageing in Africa [2, 3]. Currently, multiple myeloma is incurable. Hence, relapse after initial treatment is inevitable, leading to a condition known as relapsed/refractory multiple myeloma (RRMM).

Per definition, RRMM is preceded by at least one prior treatment. The preceding treatment(s) can either include autologous stem cell transplantation, chemotherapy or both. In South Africa, there is limited accessibility to transplantation, especially for patients who live further away from treatment centres [4]. For the past 21 years, novel chemotherapy drugs such as lenalidomide and bortezomib have been the global standard of care for treatment of RRMM patients [5–9]. These drugs have demonstrated efficacy in improving the median survival of relapsed patients, however, they are also expensive [10]. Owing to the high procurement costs, HICs are the main benefactors of these drugs with limited public funding in many LMICs [11].

Several studies have demonstrated the efficacy of lenalidomide based therapy in RRMM patients, particularly lenalidomide combined with high dose dexamethasone (LEN/DEX) [12, 13]. Based on findings in the RRMM multi-centre trials MM-009 and MM-010, the clinical effectiveness of LEN/DEX is superior to dexamethasone (DEX), which is the current standard of care in South Africa [13]. Similarly, bortezomib monotherapy (BORT) is also shown to be more effective than dexamethasone [14]. However, despite the demonstrated efficacy of the lenalidomide-based and bortezomib-based regimens, their high cost has limited access to the treatments in low and middle income countries (LMICs) like South Africa (SA), where this study is based [15].

The costs of both BORT and LEN/DEX are significantly higher than that of DEX. At single exit price (the regulated price in South Africa’s private sector pharmaceutical market) the costs of 6 cycles of treatment of LEN/DEX and BORT per patient are R364 688.75 and R729 288.00, respectively [16]. To put this in context, the average annual formal sector salary in South Africa is R282 312, in a country with a substantial informal sector and an unemployment rate of 34.4% [17]. These prices, however, do not take into consideration the benefits from each treatment. Thus, an economic evaluation was requested from the National Department of Health (NDoH) Essential Drugs Program (EDP) to analyse the treatments in terms of both costs and benefits. The use of economic evaluations is particularly valuable in settings with severely strained resources such as South Africa.

The main aim of the analysis was to conduct a cost-utility analysis of dexamethasone compared with bortezomib and lenalidomide-based regimens for the second-line treatment of multiple myeloma from a South African public health perspective. In addition, budget impact analysis was conducted to estimate the effect that introducing BORT and LEN/DEX would have on the budget of the South African public health care system. The results of the study were intended to inform the decision-making process of the body responsible for formulating treatment guidelines for the public health system in South Africa, the National Essential Medicines List Committee (NEMLC).

## Methods

We conducted a cost-utility analysis to evaluate the long-term costs and outcomes of treating a hypothetical cohort of South African patients with relapsed/refractory multiple myeloma (RRMM). The study represents a hypothetical tertiary hospital setting in South Africa where RRMM patients receive treatment. The baseline sociodemographic and clinical characteristics of the simulated population were obtained from the APEX, MM-009 and MM-010 trials [13, 14, 18]. The three trials have been described in detail elsewhere [13, 14, 18].

In summary, the APEX trial investigated the efficacy of Bortezomib in the treatment of RRMM relative to high-dose dexamethasone for participants/patients who had received one to three prior treatments [13]. The participants were randomly assigned to either the BORT or the DEX arm. Each arm received 1.3 mg⁄m2 of Bortezomib intravenously or 40 mg of Dexamethasone orally, respectively [18]. The median survival and overall response rates for the BORT arm were found to be 29.8 months and 43%, respectively; while those of the DEX arm were 23.7 months and 9%, respectively. The time-to-progression (TTP) was 6.2 months versus 3.5 months for the DEX arm [18].

The MM-009 and MM-010 phase III trials involved the recruitment of patients who were randomly assigned to either the LEN/DEX arm or the DEX arm. The difference between the two trials is the location of patient recruitment. For both trials, participants in the LEN/DEX arm received 25 mg of oral lenalidomide and 40 mg of oral dexamethasone daily for 21 days in a 28 day cycle. Participants in the DEX arm received 40 mg of oral dexamethasone once daily on days 1–4, 9–12 and 17–20 in a 28 day cycle for the first 4 cycles; thereafter, dexamethasone is administered on days 1–4 of the remaining 28 day cycles. The median survival and overall response rates for the LEN/DEX arm were found to be 38 months and 60.6%, respectively; while those of the DEX arm were 31.6 months and 21.9%, respectively. The time-to-progression for the LEN/DEX arm was 13.6 months versus 4.6 months for the DEX arm [13].

### Costs

The treatments evaluated were dexamethasone 40 mg monotherapy (DEX), bortezomib 1.3 mg/m^2^ monotherapy (BORT) and lenalidomide 25 mg + dexamethasone 40 mg (LEN/DEX). The estimated treatment costs for each regimen comprised RRMM drug costs; outpatient costs associated with drug administration and dispensing; and cost per inpatient day associated with hospital stays due to adverse drug reactions. The drug costs were obtained from government tender prices and from the database of medicine prices [25]. There was no cost available for oral dexamethasone in South Africa as it is not a registered drug. Thus, we assumed the cost of oral dexamethasone would be similar to that of its drug class member prednisone. Additionally, we also assumed a 40% price reduction on the Single Exit Prices for private sector drugs unavailable on the South African public health sector tender system, following the recommendations of the HTA methods guide for converting private sector to public sector prices [19]. The cost per outpatient visit was obtained from the SA uniform patient fee schedule (UPFS) for full fee paying oncology patients in public sector facilities [26]. The cost per inpatient day was obtained from the MOSAIC study which estimated the cost based on the Health Systems Trust District Health Barometer (12th Edition—2016/17) datafile. The barometer provides estimates of average cost per patient-day equivalent (PDE) for all public sector hospitals. In this study, we used the PDE costs for tertiary hospitals and inflated them to 2021 prices.

In order to estimate the average cost per patient for each treatment arm, the costs of outpatient visits and inpatient days were multiplied by the average utilisation rates of health services. The rate according to each treatment regimen was obtained from utilisation patterns observed in patients on second-line treatment in France [27] (Table 1). These rates are associated with the treatment of adverse events in patients.

**Table 1:** Model input parameters

Input	Base case estimate	Source
Utilisation rates per 4-week cycle		
Inpatient days DEX	0.02	[27]
Inpatient days BORT	0.0158	[27]
Inpatient days LEN/DEX	0.0125	[27]
Outpatient visits DEX	**0.3875**	[27]
Outpatient visits BORT	0.5025	[27]
Outpatient visits LEN/DEX	0.3325	[27]
DEX units used	480 mg (first 4 cycles) 160 mg (cycle 5 onward)	[28]
BORT units used	13.5 mg (first 8 cycles) 9 mg (cycle 9 onward)	[28]
LEN/DEX unit used	525 mg	[28]
Unit costs		
Cost per inpatient day	R 4 515.80	[29]
Cost per outpatient visit (NIC)	R 800.00	[26]
Cost per outpatient visit (IC)	R 2 837.00	[26]
Cost of DEX 40 mg	R 6.28	[25]
Cost of LEN 25 mg	R 1 340.03	[16]
Cost of BORT 1 mg/ml	R 865.47	[16]

*p/c* per cycle, NIC non-infusion chemotherapy, *IC* infusion chemotherapy

### Markov model

**Fig. 1:**
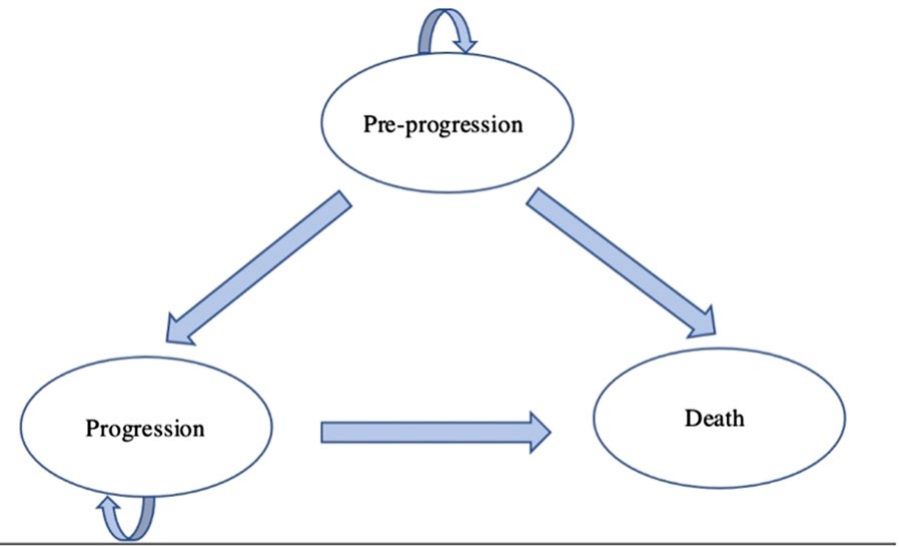
Structure of Markov model

A Markov model was used to determine the costs and outcomes of the alternate treatment regimens as shown in Fig. 1 below. The Markov states were pre-progression, progression and death. It was assumed that patients would enter in the pre-progression state. All patients in this state were assumed to have received thalidomide as first line therapy and to have stable disease on treatment with either DEX, BORT or LEN/DEX. In each cycle, the patients either stay in the pre-progression state or transition to the progression state or to dead. The progression state was characterised by a set of clinical criteria that indicate progressive RRMM disease in patients. The criteria were obtained from the MM-009/010 clinical trials [13].

Each cycle of the model was of 28 days’ duration and 195 cycles were run, generating a time horizon of 15 years in order to reflect the maximum life span of RRMM patients, given a median age at entry to the model of 63 years. The likelihood of a patient staying or transitioning to another state is determined by transition probabilities. The transition probabilities for all states in the LEN/DEX treatment arm were obtained from literature. According to the results reported by NICE (2019), these transition probabilities change with time. Time dependent transition probabilities were therefore implemented with changes in transition probabilities after 6 months and after 26 months [5]. The transition probabilities of the DEX and BORT arms were calculated by multiplying the LEN/DEX probabilities by the respective hazard ratios of the comparator treatments relative to LEN/DEX (Table 2), [5, 30]. It was assumed that the transition probabilities for these two treatments also changed at the same time points as those of LEN/DEX. While the modelled time horizon is an extrapolation beyond the period of actual observed follow up in the APEX and MM-009/010 trials, the modelling format enables extensive sensitivity analyses to be performed [31].

**Table 2:** Monthly transition probabilities of health states for patients on RRMM treatment [5, 30]

DEX transition probabilities									
Transition from	Transition to								
	0 < *t* $\le$ 6			6 < *t* $\le$ 26			26 < *t* $\le$ infinity		
	Pre-prog	Prog	Dead	Pre-prog	Prog	Dead	Pre-prog	Progr	Dead
Pre-prog	0.80	0.19	0.02	0.86	0.14	0.00	0.92	0.08	0.00
Prog	0.00	0.88	0.12	0.00	0.95	0.05	0.00	0.94	0.06
Dead	0.00	0.00	1.00	0.00	0.00	1.00	0.00	0.00	1.00

BORT transition probabilities									
Transition from	Transition to								
	0 < *t* $\le$ 6			6 < *t* $\le$ 26			26 < *t* $\le$ infinity		
	Pre-prog	Prog	Dead	Pre-prog	Prog	Dead	Pre-prog	Progr	Dead
Pre-prog	0.88	0.10	0.01	0.92	0.08	0.00	0.96	0.04	0.00
Prog	0.00	0.90	0.10	0.00	0.96	0.04	0.00	0.95	0.05
Dead	0.00	0.00	1.00	0.00	0.00	1.00	0.00	0.00	1.00

*LEN/DEX transition probabilities*									
Transition from	Transition to								
	0 < *t* $\le$ 6			6 < *t* $\le$ 26			26 < *t* $\le$ infinity		
	Pre-prog	Prog	Dead	Pre-prog	Prog	Dead	Pre-prog	Progr	Dead
Pre-prog	0.92	0.07	0.01	0.95	0.05	0.00	0.97	0.03	0.00
Prog	0.00	0.92	0.09	0.00	0.97	0.03	0.00	0.96	0.04
Dead	0.00	0.00	1.00	0.00	0.00	1.00	0.00	0.00	1.00

*Pre-prog* pre-progression, *Prog* progression, *t* time period in months

Each Markov state was associated with a cost and health outcome. The costs were measured in 2021 South African Rands and the outcomes were measured in terms of quality adjusted life years (QALYs). Health related quality of life (HRQoL) values for patients with multiple myeloma were taken from the literature [32] given that South African estimates are not available. These values are weights that quantify the preference for particular health outcomes on a scale of 0 to 1, where 0 represents poor health or death and 1 represents perfect health [31] (Table 3).

**Table 3:** Utility values for Markov states

Health state	Utility value	Source
Pre-progression	0.81	Van Agthoven et al*.* [32]
Pre-progression after 2 years	0.77	Van Agthoven et al*.* [32]
Progression	0.64	Van Agthoven et al*.* [32]
Dead	0	

*HRQoL* health-related quality of life

### Study analysis

The analysis was conducted using TreeAge Pro Healthcare 2021 software (TreeAge Pro 3 2021, R1.1, TreeAge Software, Williamstown, MA, USA; http://www.treeage.com).

The analysis follows the reference case of the South African “Health Technology Assessment Methods Guide” [19], with total costs from time of relapse to death estimated from a public health perspective, outcomes expressed as Quality Adjusted Life Years (QALYs), a 5% discount rate on costs and outcomes and findings presented in 2021 South African Rands. Where necessary, we inflated values to 2021 prices using the relevant consumer price index [20]. We used an average annual inflation rate of 5.1% and USD/ZAR exchange rate of 14.67 for the period of January-August 2021 [20]. Incremental cost-effectiveness ratios (ICERs) were estimated as the ratio of the difference in costs to the difference in QALYs between two alternative treatment regimens, with treatments ordered from least to most costly. The ICER was then compared with a range of published South African cost-effectiveness thresholds (CET) which is an estimate of the marginal productivity of the healthcare system and reflects the “health opportunity cost of health spending” in the public sector in South Africa [7, 17 p2, 18].

The hypothetical cohort of patients used in the study were assumed to be in a non-transplant setting where they are either ineligible for transplant or they do not have access to transplant services [25]. Only costs that result in differences between the three treatment regimens were included; that is, costs associated with treating comorbidities were excluded because they are assumed to be the same across the three treatment arms.

### Budget impact analysis

A budget impact analysis (BIA) was conducted from the SA public health perspective representing the annual expected financial consequences of the introduction of BORT and LEN/DEX in the public health system as well as the estimated cost of the standard of care, DEX. We estimated the percentage increase in budget impact using the cost difference between the proposed interventions (BORT and LEN/DEX) and the current regimen (DEX).

The target population was RRMM patients who had one prior treatment and were in a non-transplant setting. The patient population size was obtained from the National Cancer Registry [33] and adjusted to reflect the number expected to utilize the public health system. We included all patients from the registry because multiple myeloma is a chronic condition and relapse is inevitable. Hence, we estimated that all 337 patients would require RRMM treatment in one year and would be the target population in this analysis. The total one year costs included pharmaceutical costs and other disease-related costs. The annual outcomes were presented in terms of drug, outpatient and inpatient costs.

Given the relatively high clinical effectiveness of BORT and LEN/DEX compared to DEX, for each regimen in the base case analysis, we assumed an uptake rate of 100% which covers 100% of the RRMM population in the first year. We assumed that only one of the regimens, either BORT or LEN/DEX, would be integrated into the South African health system for RRMM patients because of current resource constraints. Therefore, we used the total population of 337 RRMM patients to estimate the budget impact for each of the three regimens.

### Sensitivity analysis

Deterministic sensitivity analyses were used to assess the robustness of the results in the CEA and the BIA. The two main sources of uncertainty in the study were identified as input data and generalisability of the results. The uncertainty of the input data was associated with transition probabilities, inpatient and outpatient costs, discount rate and drug prices. These parameters were varied and their effect on the ICER was observed. As there were no upper and lower limits for transition probabilities and inpatient and outpatient costs published in the literature, a variation of $\pm 50\%$ was done as per the SA HTA guideline [19]. However, for the transition probabilities, we capped the highest probability at 1 regardless of the result from the + 50% variation. The discount rates were varied between 0 and 10% for the analysis as per the HTA methods guide [19]. We varied the drug prices according to values found in the literature [34]. Additionally, we conducted threshold analyses on the most sensitive parameter. Finally, we conducted a scenario analysis where we evaluated the ICER of LEN/DEX versus DEX (i.e. excluding BORT). The purpose of the scenario analysis was to determine the impact on the ICER of comparing LEN/DEX directly to DEX.

The sensitivity analysis of the BIA included the variation of: drug prices, uptake rates and the target population size as these were identified as the main sources of uncertainty in the analysis.

## Results

### Base-case analysis: cost-effectiveness of DEX, BORT and LEN/DEX

Table 4 presents a summary of the cost-effectiveness results. At baseline, the per patient cost of second-line RRMM treatment using DEX, BORT and LEN/DEX is R8 312, R234 996 and R1 135 323, respectively. According to the model, the expected QALYs associated with treatment are 1.14, 1.49 and 2.22, respectively. The incremental cost effectiveness ratio of BORT versus DEX is R 654 649.00 per QALY gained while that for LEN/DEX versus BORT is R1 225 542 per QALY gained.

**Table 4:** Cost-effectiveness results of base case

Treatment regimen	Total costs	QALYs	Incremental cost per QALY gained
DEX (SOC)	R 8 312	1.14	
BORT	R 234 995	1.49	R 654 649
LEN/DEX	R 1 135 323	2.22	R 1 225 542

### Sensitivity analysis: cost-effectiveness of DEX, LEN/DEX and BORT

**Fig. 2:**
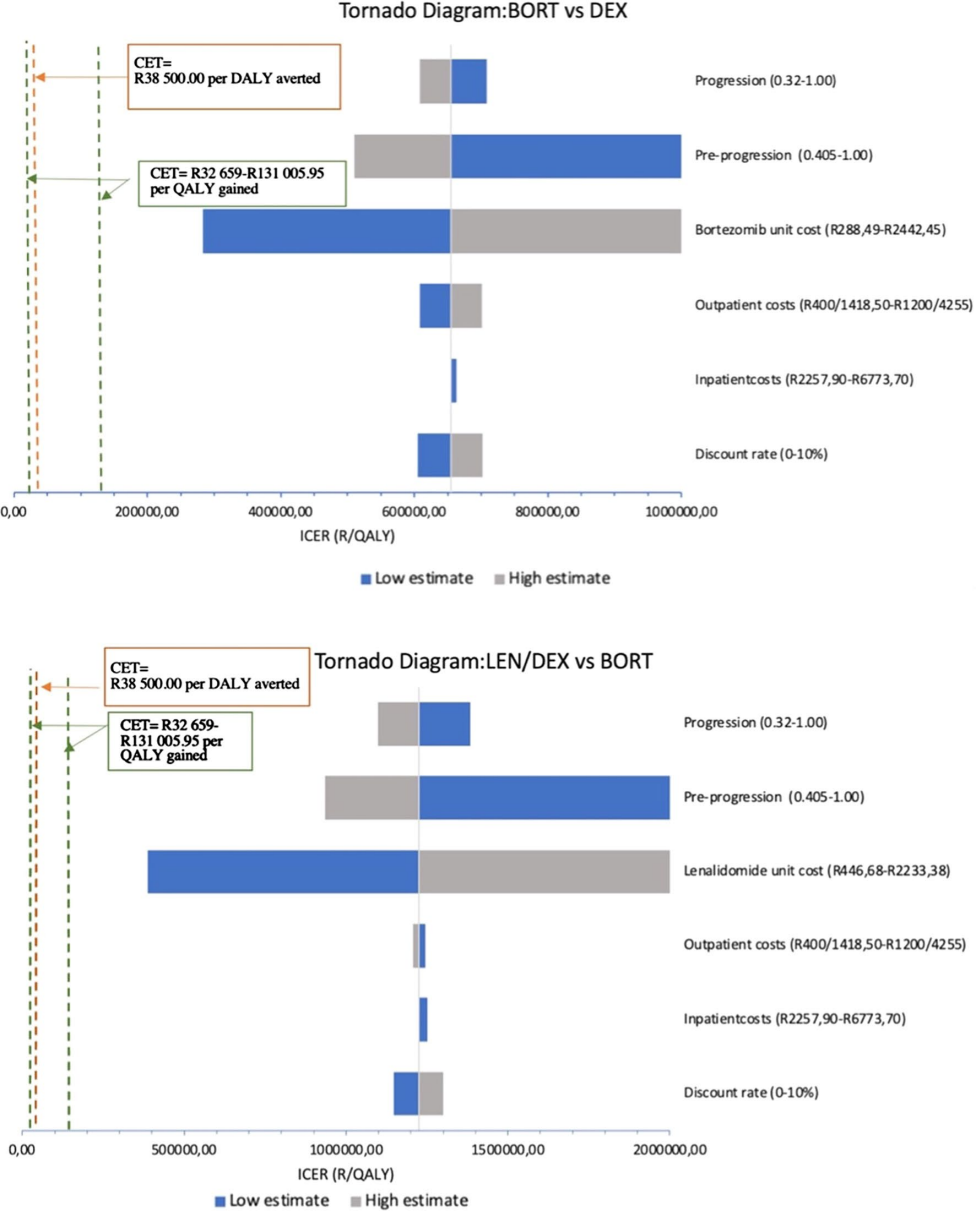
Tornado diagrams of sensitivity analyses of BORT vs DEX and LEN/DEX vs BORT

The results of the sensitivity analyses are summarised in Table 5 below. The tornado diagram in Fig. 2 shows the changes in ICERs generated by all the sensitivity analyses conducted for BORT and LEN/DEX. We interpreted the analyses by comparing the results to: the base case costs and ICERs and the cost effectiveness thresholds of R38 500 per DALY averted and R32 659-R131 006 per QALY gained [22, 23].

**Table 5:** Sensitivity analysis of DEX, BORT and LEN/DEX: costs and cost-effectiveness ratios

Sensitivity	Treatment	Cost (R)	ICER (cost/QALY)
Discount rate 0%	DEX	R 9001	
	BORT	R 257 906	605 569
	LEN/DEX	R 1 312 357	1 147 418
Discount rate 10%	DEX	R 7 741	
	BORT	R 216 818	702 510
	LEN/DEX	R 1 003 823	1 299 670
Outpatient costs − 50%	DEX	R 4 960	
	BORT	R 215 460	607 912
	LEN/DEX	R 1 130 014	1 244 907
Outpatient costs + 50%	DEX	R 11 664	
	BORT	R 254 531	701 385
	LEN/DEX	R 1 140 632	1 206 177
Inpatient costs − 50%	DEX	R 7 598	
	BORT	R 234 393	654 972
	LEN/DEX	R 1 134 750	1 225 582
Inpatient costs + 50%	DEX	R 9 027	
	BORT	R 235 598	654 325
	LEN/DEX	R 1 135 897	1 225 502
LEN drug price − 40%	DEX	R 8 312	
	BORT	R 234 996	Dominated
	LEN/DEX	R 386 478	349 861
LEN drug price + 40%	DEX	R 8 312	
	BORT	R 234 996	654 649
	LEN/DEX	R 1 884 168	2 244 883
BORT drug price − 40%	DEX	R 8 312	
	BORT	R 96 891	279 817
	LEN/DEX	R 1 030 119	1 402 216
BORT drug price + 40%	DEX	R 8 312	
	BORT	R 589 737	Dominated
	LEN/DEX	R 1 135 323	742 662

The cost of DEX remains the lowest of all three treatment regimens in all sensitivity analyses, followed by BORT then LEN/DEX. The analyses show that the cost-effectiveness of both BORT and LEN/DEX are sensitive to variation in the HRQoL associated with the pre-progression Markov state and a variation in the respective drug costs. For both regimens, decreases in the drug costs generate ICERs closest to the range of estimated CETs for the South African public health sector. Thus, the results indicate that the introduction of BORT and LEN/DEX treatment would likely be cost-effective interventions if the drug prices were lower. Our results suggest that even at the highest HRQoL of 1, both treatments would not generate ICERs close to the SA CETs.

We conducted a threshold analysis to estimate the prices of bortezomib and lenalidomide which would render the two regimens cost-effective relative to the range of CETs found in the literature. If we assumed the highest CET of R131 006 per QALY gained, we found that the baseline price of BORT would need to be reduced by 93%. Similarly, using the same CET, we found that the price of lenalidomide would need to be reduced by 96%. Higher price decreases would be needed to generate cost-effective ICERs if the lower CETs in the literature were considered.

Finally, the scenario analysis excluding BORT generated a slightly improved ICER of R1 042 656 per QALY gained for the LEN/DEX strategy versus DEX. The improvement is largely due to the larger difference in QALYs gained from LEN/DEX compared to DEX as opposed to when compared to BORT.

### Base-case analysis: budget impact of DEX, BORT and LEN/DEX

The burden of multiple myeloma (MM) in the South African population was estimated to be 337 individuals in 2016 [33]. We estimated the cost of the standard of care DEX as a baseline to quantify the additional expected cost if BORT or LEN/DEX regimens were included in the public health sector. As shown in Table 6 below, the cost of therapy annually for 337 patients on DEX is R1 373 821 and after the inclusion of BORT and LEN/DEX costs increase to R44 461 215 and R120 674 625, respectively. The main cost drivers in both LEN/DEX and BORT regimens are pharmaceutical costs. Nevertheless, we noted that BORT had significantly higher non-pharmaceutical costs compared to DEX and LEN/DEX owing to its higher hospital utilisation rates.

**Table 6:** Base case analysis: 1-year budget impact analysis of DEX, BORT and LEN/DEX

Treatment regimen	Pharmaceutical costs	Non-pharmaceutical costs	Total costs	Percentage increase from SOC
DEX (SOC)	R 39 877	R 1 333 944	R 1 373 821	
BORT	R 38 805 287	R 5 655 928	R 44 461 215	3136%
LEN/DEX	R 119 485 065	R 1 189 559	R 120 674 625	8684%

### Sensitivity analysis: budget impact of BORT and LEN/DEX

The sensitivity analysis results indicate that the total costs of both BORT and LEN/DEX are sensitive to all parameters that were varied. As seen in Table 7 below, the highest percentage variation was observed on the drug price parameter.

**Table 7:** Sensitivity analysis: 1-year budget impact analysis of DEX, BORT and LEN/DEX

Sensitivity parameter	Total costs	
	Low estimate (% change from base case)	High estimate (% change from base case)
Unit cost: LEN/DEX (R446,68-R2233,38) BORT (R288,49-R2442,45)	R 41 047 217 (−66%)	R 200 302 026.11 (+ 66%)
	R 16 564 932 (−63%)	R 97 981 213.28 (+ 120%)
Population coverage: LEN/DEX (50%-80%) BORT (50%-80%)	R 48 269 850 (−60%)	R 96 539 700 (+ 20%)
	R 17 784 486 (−60%)	R 35 568 972 (+ 20%)
Uptake rate: LEN/DEX (50%) + DEX (50%)	R 48 819 379 (−60%)	R 96 814 464 (+ 20%)
BORT (80%) + DEX (20%)	R 18 334 015 (−59%)	R 35 843 737 (+ 19%)

## Discussion

Our analysis suggests that, based on the base case values and potential cost-effectiveness thresholds of R32 659, R38 500 and R131 006, neither BORT nor LEN/DEX are cost-effective second-line therapies for RRMM patients in SA. Nevertheless, BORT was considerably more cost-effective and had less impact on the healthcare budget than LEN/DEX. Although LEN/DEX is the most effective second line treatment for RRMM patients in this analysis, the additional costs associated with the incremental benefit of the treatment render it cost-ineffective. Similarly, the scenario analysis showed that even when compared with DEX as a standard of care (assuming no availability of BORT), the ICER of LEN/DEX was above any of the CETs that we considered. The main cost driver was the price of lenalidomide, and as shown in the sensitivity analysis, an extremely high price decrease would be needed to bring LEN/DEX into the cost-effective range. Given these findings, we suggest that the best value for the SA government would be to add BORT as a second-line treatment option for non-transplant RRMM patients provided that the drug price is decreased and the increased budget is affordable on an equitable basis.

The study findings were consistent with the findings of Cai et al*.* [35], Hornberger et al. [36] and Liwing et al. [37] who reported that BORT was relatively more cost-effective than LEN/DEX [35–37]. These studies were conducted from Chinese, Swedish and Nordic (Swedish, Danish and Norwegian) perspectives, respectively. We noted that there was a contradiction in findings from studies with similar perspectives. Möller et al. [38] found that LEN/DEX was cost-effective from a Norwegian perspective while Liwing et al. [37] reported that it was not [37, 38]. We attribute the difference in results to the methodological differences and survival assumptions of the two studies.

In contrast, the results of our analysis were dissimilar with studies that have been conducted in other settings. Six studies conducted in Chile, the United Kingdom (UK), South Korea and Norway recommended that LEN/DEX was cost effective when compared with Bort [38–43]. Notably, all these studies were conducted in middle and/or high income countries where cost-effectiveness thresholds are higher than in South Africa.

Interestingly, even though all the aforementioned countries are HICs with relatively higher fiscal capacities, the high price of lenalidomide was a main cost driver which negatively impacted the ICERs in such countries. In order to overcome this in the UK, a cost reduction agreement referred to as “the patient access scheme” was made with the pharmaceutical company Celgene [5]. A study by Wouters et al*.* [44] provides evidence of the SA department of health also being able to procure drugs at lower costs. Thus, we suggest it is possible for a price reduction of BORT and/or LEN/DEX to be made in the SA context [44]. According to Wouters et al*.* [44], during the period of their study, drugs were procured at low prices in the public health sector such that they cost considerably more in the private sector [44]. Additionally, the study also found that over time, the public sector prices further decrease due to procurement via a tender system. In particular, the average prices of all drugs and oncology drugs on tender decreased by 40% and 70%, respectively, between 2003 and 2016 [44].

Our study findings were consistent with those of the Health Intervention and Technology Assessment Programme (HITAP) in Thailand which reported that for LEN/DEX to be cost-effective, the price of lenalidomide would need to be reduced by 98% [7]. In addition, they also found that BORT was not cost-effective in the Thai context even when the bortezomib drug price was reduced [7]. Similarly, in the United Kingdom and Canada, LEN/DEX for RRMM is only recommended to patients on condition that it is provided under patient access schemes, which improves the relative cost-effectiveness of the treatment [5, 6]. In Australia and New Zealand LEN/DEX is recommended “on a cost-minimisation basis” with BORT where the two treatments are provided at equi-effective doses [43 p7, 44]. Our study illustrates how high prices of cancer drugs can be a major barrier to access. In particular, LMICs are severely impacted due to poor procurement policies and sustained patents for the more expensive originator drugs [45, 46].

Cost-utility analyses are useful tools in decision-making in the healthcare sector as they give an indication of which interventions offer the best value for money. However, these analyses do not provide information on affordability or budget impact of the interventions [47]. Thus, we conducted a budget impact analysis to assess the impact BORT and LEN/DEX would have on the Department of Health budget. We found that both BORT and LEN/DEX significantly increase the cost of RRMM treatment—by 3136% and 8184% respectively, largely due to pharmaceutical costs. It was noted that LEN/DEX had significantly lower non-pharmaceutical costs than BORT which aligns with findings from Elsisi et al*.* [48] who conducted a similar analysis in Egypt [48].

We attributed the difference in non-pharmaceutical costs between BORT and LEN/DEX to the increased hospitalisation and outpatient visits associated with BORT relative to LEN/DEX. In accordance with our findings, one study showed that lenalidomide can be cost saving because it reduces the risk of hospitalisation due to adverse effects and complications [28]. Similarly, a study by Durie et al*.* [49] also reported that LEN/DEX was budget saving compared to BORT with regards to administrative costs [49]. These findings suggest that when compared to BORT, LEN/DEX has the potential to be budget-saving to the provider if the pharmaceutical costs are reduced. However, given that in SA the current standard of treatment is DEX, our study findings suggest that the budget-saving benefits of LEN/DEX would only be limited to a small margin saving on non-pharmaceutical costs while pharmaceutical costs would be considerably high.

There are several limitations to this study mainly due to underlying assumptions to address local data availability. First, the inputs of the model relied on secondary data from clinical trials that were conducted in Europe and Israel. Consequently, we assumed South African RRMM patients would have similar characteristics as APEX and MM-009/010 patients, which may not be the case. Similarly, data regarding the utilisation of healthcare services in RRMM patients in South Africa were unavailable; hence the European estimates used in this study are subject to uncertainty in the SA context. Third, the effectiveness data were obtained from three different clinical trials which necessitated an indirect comparison of the drugs. However, we made similar assumptions as were made in other CUAs.

Due to lack of data about age-specific death rates in SA, our analysis did not consider background mortality of RRMM patients in the country; that is, we did not include the probability of dying from other causes for the patients included in the analysis. To account for this limitation, we limited the time horizon of the model to 15 years. Finally, the analysis did not consider the impact of co-morbidities on the patient pathway nor the reduction in HRQoL associated with adverse events from BORT and LEN/DEX. The exclusion of these considerations was due to a lack of data availability.

## Conclusion

This analysis has shown that at current drug prices, both BORT and LEN/DEX would not be cost-effective strategies for second-line treatment of RRMM patients in South Africa. The budget impact of both drugs is substantial and careful thought should be given to issues of equitable access. However, based on evidence from other settings and our study, BORT would potentially be cost-effective and affordable for the SA government budget if the price of the drug were to be reduced. Hence, these findings support the consideration of BORT treatment for RRMM patients in South Africa and are likely to be applicable in other LMICs with constrained health budgets.

## Data Availability

Not applicable.

## Abbreviations

BIA: Budget Impact Analysis
Bort: Bortezomib
CEA: Cost-Effectiveness Analysis
CUA: Cost utility analysis
HIC: High income country
HRQoL: Health-Related Quality of Life
ICER: Incremental Cost Effectiveness Ratio
ICUR: Incremental Cost Utility Ratio
LEN/DEX: Lenalidomide + low-dose dexamethasone
LMIC: Low-middle income country
MM: Multiple myeloma
QALY: Quality-Adjusted Life Year
RRMM: Relapsed/Refractory multiple myeloma
SA: South Africa
SOC: Standard of care
